# Seeds of change: First assessment of an interprofessional training for medical and nursing students through INITIAL (“INnovative InTerprofessIonAl Learning in primary care”): A mixed-method evaluation

**DOI:** 10.3205/zma001813

**Published:** 2026-02-17

**Authors:** Melanie Mauch, Jessica Kauffmann, Marlene Berger, Cornelia Mahler, Nadine Röhrig, Hannah Fuhr, Friederike Schalhorn, Roland Koch, Olaf Fritze, Sylvia Schrempf, Heidrun Sturm

**Affiliations:** 1 University Hospital Tübingen, Institute of Health Sciences, Nursing Science, Tübingen, Germany; 2 University Hospital Tübingen, Institute of General Practice & Interprofessional Healthcare, Tübingen, Germany; 3 University of Tübingen, Tübingen Institute for Medical Education (TIME), Tübingen, Germany

**Keywords:** education, interprofessional collaboration, healthcare team, program evaluation

## Abstract

**Objective:**

Staff shortages create an urgent need for enhanced interprofessional collaboration (IPC). Interprofessional education (IPE) prepares healthcare professionals to address such challenges. In this study we evaluated an IPE seminar conducted in the winter semester of 2023.

**Method:**

We used the University of West of England Interprofessional Questionnaire (UWE-IP-D) and the Interprofessional Socialization and Valuing Scale (ISVS-9A/9B) to assess shifts in students' attitudes towards IPC. Assessments were carried out before and after the seminars, supplemented by qualitative feedback from the students.

**Results:**

21 participants (7 nursing, 14 medical; average age of 24.5 years (range 19-35years)) completed both the evaluations. Initial assessments suggest positive attitudes towards teamwork and interprofessional learning. The post-seminar results showed improved communication and teamwork scores. Further improvements were observed in interprofessional socialization and valuing. Qualitative feedback pointed to potential areas for improvement.

**Conclusion:**

The results showed positive attitudes towards IPC. The study was limited by the number of participants, the lack of a control group and the brief duration of the seminar (two days). Nevertheless, we will incorporate student feedback into future iterations of the INITIAL seminar as well as in other IPE activities.

## Zusammenfassung

**Zielsetzung:**

Angesichts des Fachkräftemangels gewinnt die interprofessionelle Zusammenarbeit (Interprofessional Collaboration, IPC) zunehmend an Bedeutung. Interprofessionelle Ausbildung (Interprofessional Education, IPE) befähigt Gesundheitsfachpersonen dazu, diesen Herausforderungen besser zu begegnen. In dieser Studie wurde ein IPE-Seminar evaluiert, das im Wintersemester 2023 durchgeführt wurde.

**Methode:**

Zur Erfassung von Einstellungsveränderungen der Studierenden gegenüber IPC kamen der „University of West of England Interprofessional Questionnaire“ (UWE-IP-D) sowie die „Interprofessional Socialization and Valuing Scale“ (ISVS-9A/9B) zum Einsatz. Die Erhebungen fanden vor und nach dem Seminar statt und wurden durch qualitative Rückmeldungen der Studierenden ergänzt.

**Ergebnisse:**

Insgesamt 21 Studierende (7 Pflegestudierende, 14 Medizinstudierende; Durchschnittsalter 24,5 Jahre [Bereich 19–35 Jahre]) nahmen an beiden Erhebungen teil. Bereits die Eingangsbefragung zeigte positive Haltungen gegenüber Teamarbeit und interprofessionellem Lernen. Nach Seminarende zeigten sich Verbesserungen in den Skalen zu Kommunikation und Teamarbeit. Zusätzliche Fortschritte ergaben sich in den Bereichen interprofessionelle Sozialisation und Wertschätzung. Das qualitative Feedback identifizierte potenzielle Ansatzpunkte zur Weiterentwicklung.

**Schlussfolgerung:**

Die Ergebnisse weisen auf positive Einstellungen gegenüber IPC hin. Limitiert wird die Studie durch die geringe Anzahl an Teilnehmenden, das Fehlen einer Kontrollgruppe sowie die kurze Dauer des Seminars (zwei Tage). Gleichwohl werden die Rückmeldungen der Studierenden in die Weiterentwicklung des INITIAL-Seminars und anderer IPE-Angebote einfließen.

## Introduction

The rising prevalence of multimorbidity and the shortage of medical and nursing staff, driven by demographic changes, place increasing pressure on primary care as the first point of contact in healthcare systems across Europe [1], [2]. As the first point of contact for patients, primary care must respond to complex, long-term needs while maintaining continuity and accessibility. Yet, care delivery often becomes fragmented, with detrimental effects on quality, coordination, and patient outcomes. 

Interprofessional collaboration (IPC) is widely recognised as a key strategy to address these challenges. It facilitates shared responsibility, holistic care planning, and efficient use of resources, particularly in the management of chronic diseases, post-hospital transitions, and in supporting patients who lack informal carers or whose families require assistance. IPC has been shown to improve clinical outcomes, reduce preventable hospital admissions, and enhance overall system performance [3], [4], [5], [6]. With rising demand due to demographic shifts and professional shortages, there is a growing consensus that IPC must become an integral component of high-quality primary care [7], [8]. However, implementation remains limited, often hindered by structural barriers such as rigid professional boundaries, lack of shared time, and insufficient communication infrastructure [3]. 

Interprofessional education (IPE) promotes IPC and thus plays an important role in overcoming these obstacles. By enabling health professionals to learn with, from and about each other, IPE fosters mutual understanding, role clarity, and collaborative competencies [1]. Reforming health professionals' education through IPE is therefore crucial to foster collaboration. Mulvale et al. suggest integrating IPE into curricula, providing organizational support, and using standardized communication tools to improve patient outcomes and reduce costs [9]. 

The relevance of IPC is particularly evident in primary care, where teams must respond to diverse, unpredictable and long-term patient needs. Embedding IPE within this context is therefore essential. Primary care-focused IPE initiatives, such as joint case discussions, community-based placements, or simulation of team-based consultations, have been shown to improve collaborative decision-making and reduce hierarchical barriers [3], [10], [11], [12]. They also prepare future professionals for the realities of interdisciplinary coordination in outpatient settings. 

International research underlines that IPE contributes to safer, more effective primary care. It improves teamwork and role understanding, enhances clinical confidence, and supports the development of inclusive, patient-centred services. Moreover, IPE can promote equity by ensuring that all professional groups have access to collaborative training, helping to build diverse and responsive primary care teams capable of meeting the complex needs of ageing and often underserved populations [3], [13], [14]. 

The University of Tübingen offers medical training and a Bachelor of Science in Nursing, conducted in collaboration with Esslingen University of Applied Sciences. After seven semesters, nursing students graduate with both professional certification as nursing professionals and an academic degree, immersed in an environment where IPC is a key part of the curriculum. In contrast, the medical programme lacks an explicit focus on IPE, representing a critical gap in preparing medical students for the realities of contemporary healthcare practice. The INITIAL (INnovative InTerprofessionAL Learning in Primary Care) seminar addresses this gap by fostering interprofessional understanding and collaboration. Its primary objective is to broaden future healthcare professionals' perspectives, enabling them to critically reflect on their work.

### Aim

This pre-post mixed-methods pilot study evaluated changes in students’ attitudes towards interprofessional learning, capturing their perspectives and their subjective assessments of the seminar. The central research question was: “Does the INITIAL seminar change attitudes towards interprofessional learning among medical and nursing students, and what are potential implications for their future professional practice?”.

### Framework

The design and implementation of the INITIAL seminar were guided by the six-step approach to curriculum development described by Kern et al. This structured model provides a systematic framework for identifying the educational needs of the target groups, defining IPE objectives, selecting appropriate teaching strategies, and planning evaluation procedures. Following a problem-centred needs assessment, IPE goals were derived, informed by known barriers to collaboration in primary care. Educational strategies – such as small-group case work, tandem teaching, and structured reflection – were selected to address these goals. Feedback from participants and evaluation data will be used for refinement in accordance with the model’s emphasis on continuous improvement [15]. 

## Methods

We used a mixed-method evaluation for this two-day seminar. It included two pre- and post-seminar questionnaires at T0 and T1, along with a qualitative analysis of three open-ended questions.

### Seminar

INITIAL (INnovative InTerprofessionAL Learning in Primary Care) enables medical and nursing students to study together and learn from one another. Developed in 2022 at the University of Tübingen, it focuses on IPC topics especially in complex multimorbid primary care patients. Students work in small interprofessional groups throughout the seminar. The two-day seminar (held on December 2^nd^ and 16^th^, 2023) consisted of 16 on site teaching units (45 minutes each) and four online Q&A sessions. Theoretical background lectures on interprofessional theories and IPC in literature and studies, along with medical and nursing care content (especially diabetes and diabetic foot ulcer) were alternated with interprofessional group studies. Lectures were delivered by teaching tandems comprising medical doctors and nurse researchers. 

Before the first group session an interactive ice-breaker game was used to introduce participants to each other. The students worked in small interprofessional groups on their case study on an elderly patient with diabetes mellitus in a complex social and medical context. On the second day, after discussing IPC in primary care in Germany as well as IPC in research an interprofessional case presentation of the groups focused on the medical and nursing care plan for this patient that were co-developed by both student groups.

### Participants and procedure

Nursing students attended INITIAL as a mandatory part of their curriculum, while medical students chose it as an elective during their clinical phase. The elective was promoted through posters and the intranet platform. Students were informed about the study’s content and purpose, provided with an information sheet, data protection details, and a consent form, which they signed before completing the electronic evaluation forms accessed via a QR code at the seminar’s start. Participation in the evaluation was voluntary and not linked to attendance or successful completion of the seminar. 

### Questionnaires

Two scales of the *University of the West of England Interprofessional Questionnaire* (UWE-IP-D) [16] were used before (T0) and after (T1) the seminar: *communication and teamwork* (9 items) and *interprofessional learning* (9 items). Additionally, the short Version of the *Interprofessional Socialization and Valuing Scale* (ISVS-9A and ISVS-9B) [17], [18] was used to evaluate shifts in interprofessional attitudes. Permission to use both scales was obtained.

### University of the West of England interprofessional questionnaire (UWE-IP-D)

The UWE-IP-D was used to measure changes in the participants’ understanding and perceptions of IPE and IPC, particularly in communication and education. The UWE-IP questionnaire assesses how participants perceive the importance and effectiveness of IPC and educational collaboration.

The *communication and teamwork* scale of the UWE-IP evaluates the quality and frequency of communication between different healthcare professionals. This includes aspects such as clarity of communication, willingness to share information, and trust in the communication skills of team members. Items are rated on a 4-point Likert scale, with options ranging from 1 (strongly agree), 2 (agree), 3 (disagree), to 4 (strongly disagree). Individual item scores are summed to yield a total score ranging from 9 to 36. Scores are interpreted as follows: 9-20=positive, 21-25=neutral, and 26-36=negative attitudes toward interprofessional communication and teamwork.

The *interprofessional learning* scale focuses on how participants experience the IPE process. This includes the perceived relevance of interprofessional learning, willingness to collaborate in educational settings, and understanding of one’s own role and the roles of other professions in the educational process. Participants responded to these items on a 5-point Likert scale, with response options ranging from 1 (strongly agree), 2 (agree), 3 (neutral), 4 (disagree), to 5 (strongly disagree). Item scores were summed to produce a total score ranging from 9 to 45. Interpretation of the total score follows this categorisation: 9-22=positive, 23-31=neutral, and 32-45=negative attitudes towards interprofessional learning.

### Interprofessional Socialization and Valuing Scale (ISVS)

The German 9-Item Version (9A /9B) of the ISVS [18] was applied to measure interprofessional socialization, including the recognition, integration, and practical application of values and attitudes towards IPC in healthcare settings at T0 and T1. Participants responded to these items using a 7-point Likert scale, with options ranging from 1 (strongly disagree), 2 (disagree), 3 (somewhat disagree), 4 (neutral), 5 (somewhat agree), 6 (agree), to 7 (strongly agree), and 0 (no answer). The mean score rating was calculated using standard arithmetic procedures. This allowed for the comparison of pre- and post-seminar responses, offering insight into changes in interprofessional attitudes over time. 

### Open-ended questions

Qualitative analysis involved evaluating open-ended questions posed before and after the seminar to capture participants’ expectations (T0), their experiences during the seminar, and the applicability of learned concepts to clinical practice (T1):

- “What are your expectations for the seminar?” (T0)
- “Were your expectations for the seminar met?” (T1)
- “Take Home Message: What will you take from the seminar into your next clinical placement?” (T1)
- “Is there anything else you would like to share with us?” (general feedback) (T1)

### Data collection 

All seminar participants were invited to the study. Data were collected electronically using the Research Electronic Data Capture (REDCap) software, managed by the Tübingen Institute of Medical Education (TIME). The database was developed to capture longitudinal data on interprofessional developments within the Medical Faculty of Tübingen. REDCap provides a secure interface for students to complete a pseudonymised survey. Participants completed the questionnaires at two time points: immediately before the seminar (T0) and immediately after the seminar (T1), accessible via QR codes. This pre-post design allowed for the assessment of changes in attitudes and perceptions resulting from the seminar.

At T0, the participants provided sociodemographic information, including age, gender, and educational background. This information was used to describe the participant population and analyse the potential demographic influences on the outcomes. To protect the participants' privacy, all responses were pseudonymised using unique codes to link data across the two time points without disclosing personal identities. Data were securely stored within the REDCap system, with access restricted to authorised personnel. The system complies with EU data protection regulations and institutional research ethics. The study protocol was reviewed and approved by the Ethics Commission of the Eberhard Karls University of Tübingen (reference number 638/2023B02). Ethical considerations included voluntary participation and the right to withdraw at any time without penalty. These were addressed through written consent and an accompanying information letter provided to all participants.

### Data analysis

#### Quantitative analysis

Sum scores for the UWE-IP-D scales and the mean scores for the ISVS-9A/9B were calculated for T0 and T1. Due to the small sample size, sum scores were analysed descriptively to identify trends.

#### Qualitative analysis

The open-ended responses were independently coded and analysed by two nursing scientists according to the content analysis by Philipp Mayring [19]. Three steps were performed: paraphrasing, reduction, and summarisation. Differences in coding were resolved through discussion to ensure consistent analysis. The qualitative analysis aimed to gain a deeper understanding of the students' learning processes and capture aspects not included in the standardised questionnaires. 

## Results

### Study population characteristics

A total of 21 participants (14 medical and 7 nursing students) responded. However, an additional 7 participants (all nursing students) were periodically absent and therefore did not complete the pre- and post-evaluation. The average age was 24.5 years. Nursing students were all in their 5^th^ semester, while medical students were in semesters 6 to 9. The gender distribution was 16 females (76.2%) and 5 males (23.8%). Some medical students had prior professional experience, including nursing qualifications and training as emergency paramedics and medical-technical assistants (see table 1 (Tab. 1)). 

### Quantitative results

*UWE-IP-D:* In the *communication and teamwork* *scale*, the sum scores of all participants decreased from T0 to T1, indicating a positive development. The sum scores in the *interprofessional learning* *scale* also decreased from T0 to T1, indicating enhanced understanding and appreciation for interprofessional learning. The following table shows the change in sum scores (see table 2 (Tab. 2)).

*ISVS:* The* Interprofessional Socialization and Valuing Scale* (ISVS 9A/9B) showed an increase in mean scores from T0 to T1, indicating a more positive attitude towards interprofessional collaboration. The following table shows the changes in mean scores (see table 3 (Tab. 3)). 

### Open-ended questions

The free-text responses of the 21 students who participated in the evaluation were analysed. For the open-ended question “Expectations met”, four categories were found. Two of these categories addressed organisational matters:

1. shorter lecture times, and
2. more group work.

The following quote reflects a participant's opinion on group work: “I* found it a bit disappointing that there wasn’t much collaboration within the groups, except during the presentations.” (P38N).* The other two categories focused on content-related areas:

1. more case studies, and
2. tips for implementing IPC and dispelling stereotypes.

*“I would have expected to discuss more patient cases and also to have more practical tasks.” (P28M).* The participants anticipated a stronger emphasis on the discussion of patient cases and inclusion of more practical tasks. This feedback points to a potential opportunity to enhance seminars by incorporating more case-based discussions and practical activities.

The “take home messages” responses highlighted three key categories, which can be described as followed. Category 1 “communicate more with each other” describes the students need for enhanced communication. One participant noted,* “Communication is important, but difficult to implement due to missing structures” (P21N).* The category *“learn more from each other (knowledge)”* can be best underlined with the following quote: *“[I learnt] that we communicate with each other about what the other can or cannot do” (P24M). The category “interprofessional working improves the quality of care”* and the quote,* “That we all achieve the same goal best by exchanging information and combining nursing and medical aspects. However, the patient is at the centre of it all,” (P11N)* shows the students’ understanding of the importance of IPC and exchange between healthcare professions. 

The questions “expectations beforehand” and “is there anything you would like to share with us? (general feedback)” did not provide new information, as the answers were consistent with those from “expectations met” and “take home messages”.

## Discussion

The central question guiding this evaluation was: “Does participation in the INITIAL seminar lead to changes in attitudes towards interprofessional learning among medical and nursing students? What are potential implications for their future professional practice?” The quantitative results suggested positive developments in communication and teamwork as well as a deeper appreciation for interprofessional learning. Qualitative analysis revealed key aspects the students prefer, such as shorter lecture times, more group work, more case studies, and advice for implementing IPC. The findings suggest that the INITIAL seminar improved students’ attitudes towards IPC and learning, thereby affirming the central research question.

### Study population and characteristics

The age distribution reflects a relatively young group at the beginning of their professional careers. Heterogeneity in the background enriched interdisciplinary discussions and the exchange of experiences within the seminar. The presence of students from different stages of educational development brought a wider perspective to IPC. There were more female than male students, which corresponds to the typical gender distribution in the respective fields [20].

### Quantitative results

Descriptive analysis of the quantitative results revealed trends and differences between the two time-points of data collection. In the area of* communication and teamwork* (UWE-IP-D), the sum scores of all participants decreased from T0 to T1, indicating positive development in these areas. This suggests an improvement in the participants’ ability to communicate and work as a team. In *interprofessional learning* (UWE-IP-D), there was also a reduction in sum scores from T0 to T1, which indicates enhanced understanding and appreciation for interprofessional learning among the students. *Interprofessional socialization and valuing* (ISVS 9A/9B) showed improvement in mean scores from T0 to T1, highlighting the importance of social aspects of IPE. These findings are consistent with those of Berger et al., who evaluated an IPE seminar at Heidelberg University’s Medical Faculty, involving 132 undergraduate students (103 female, 29 male) from different professions including medicine and nursing. The course was also structured as a two-day block seminar combining lectures, patient narratives, and small-group case work. To evaluate the course’s impact, the authors administered the UWE-IP-D and statistically significant improvements were observed across all subscales, indicating that the seminar positively influenced students’ attitudes and self-perceived competencies in interprofessional collaboration [21]. 

### Qualitative results

The qualitative analysis provides insights into students’ experiences and perceptions. The responses show that students valued practical and interactive components and desired more opportunities to engage in group activities and real-world case discussions. Similar expectations were reported by Schwarzbeck et al., whose participants suggested discussing case studies and receiving practical training in emergency care and communication with patients [22]. Bridges et al. implemented an IPE seminar involving first-year students (n=480) from various health professions including medicine and nursing students. Evaluation through post-course surveys and focus groups indicated positive shifts in student attitudes towards collaboration, teamwork, and social responsibility, while also revealing continued need for structured reflection and deeper role understanding within interprofessional teams [23]. 

It can be concluded that in general IPE seminars should include a high amount of case studies and lively discussions between professionals. Students appreciate teamwork and topics relevant to clinical practice. The students’ observation regarding communication reveals the significance of communication for effective teamwork and patient care, but also the challenge of implementing it due to the lack of supportive structures. This emphasis on communication about individual competencies and limitations highlights that effective IPC goes beyond simply working alongside one another. It involves a deep understanding of the skills and expertise of each team member. The students also emphasized that the integration of nursing and medical perspectives is key to achieving optimal patient care. This approach reinforces the necessity of moving beyond the confines of individual disciplines to adopt a more integrated patient-care perspective. Fostering interprofessional exchange leads to improved quality of care via a more comprehensive understanding of the patients and their needs.

The results of our evaluation are in line with those from the review of Witt Sherman et al., who suggest that effective IPC in healthcare can be promoted through university support and faculty involvement. The review highlights the need for strategic planning and resource allocation by universities to support IPE initiatives. It emphasizes that both top-down and bottom-up approaches are necessary to create a culture of IPC and overcome challenges in terms of structure, processes, and outcomes [24]. The results of Berger et al. suggest that pragmatic approaches are essential when introducing IPE initiatives into undergraduate health science curricula. Such approaches can help to overcome institutional barriers and to demonstrate that IPE can lead to positive learning outcomes. The authors emphasize the importance of adopting mixed methods for evaluation to capture both the quantitative and qualitative aspects of IPE outcomes. The general long-term goal of IPE is to enhance IPC among health professionals, thereby improving patient safety and the quality of care [21]. 

### Limitations

The very small sample size of 21 students in a pilot setting limits the generalizability of the findings; future research should include larger, more diverse groups. The seminar’s brief two-day span restricts the assessment of long-term IPE effects on attitudes and practice, indicating a need for extended interventions. Selection bias may have influenced outcomes: participation was voluntary for medical students, who likely already held favourable views on IPC, while nursing student involvement was mandatory, possibly not reflecting genuine interest. Additionally, to avoid scheduling conflicts, the seminar was held on weekends, resulting in increased workload for participating students. For sustainable and broader implementation, adjustments to the medical curriculum are necessary. However, such changes would require a fundamental and cross-disciplinary rethinking of timetable structures to enable integrated IPE without overburdening students.

However, the absence of a control group and reliance on self-reported data challenge the ability to directly link changes to the seminar, potentially skewing results towards social desirability. Finally, although both quantitative and qualitative methods were applied, the depth of analysis – especially in qualitative feedback – could be enhanced to gain deeper insights into interprofessional learning experiences. Addressing these limitations through broader participant recruitment, longer study durations, inclusion of control groups, and methodological refinement is essential for advancing IPE research and its application in healthcare education. 

## Implications and conclusion

The findings of this mixed-methods pilot evaluation demonstrate that IPE in the context of primary care is both relevant and well received by students of medicine and nursing. The seminar created a space for collaborative exchange, where students began to understand and appreciate the perspectives, competencies, and limitations of the other profession. Quantitative results indicated positive developments in interprofessional attitudes, particularly in communication, teamwork, and learning readiness, while qualitative responses confirmed that students perceived the seminar as beneficial and applicable to clinical practice. Nevertheless, the data also highlight areas for improvement in both content and structure. Several students expressed a desire for more time allocated to case-based group work and fewer lecture units. Others suggested that IPC should be further supported through practical tasks, structured feedback, and clearer team processes. These insights suggest that future iterations of the seminar should enhance interactive elements, increase opportunities for reflective discussion, and incorporate more concrete examples of interprofessional practice. In addition, greater emphasis on the implementation of IPC in real-world outpatient settings may help students link their seminar experiences more directly to their clinical routines.

Taken together, the results suggest that structured IPE interventions can be successfully implemented in undergraduate curricula, even in short formats. To maximise their impact, however, continuous refinement based on student feedback is essential. This includes aligning seminar design more closely with students’ expectations and clinical needs and embedding IPE more firmly across both nursing and medical education pathways. The INITIAL seminar offers a foundation for such integration and can serve as a model for further development of primary care-oriented IPE at our institution.

## Acknowledgements and funding

We would like to express our gratitude to the students who participated in the seminar and the subsequent evaluation. Their commitment and contribution were essential to the success of this seminar. We also extend our gratitude to PROFILPlus of the Medical Faculty Tübingen for funding this seminar (project number F.7720010.1). 

## Authors’ ORCIDs

- Melanie Mauch: [0000-0001-7240-8388]
- Cornelia Mahler: [0000-0002-6601-0602]
- Roland Koch: [0000-0002-6500-928X]
- Olaf Fritze: [0000-0002-3825-3703]
- Heidrun Sturm: [0000-0003-4327-7205]

## Competing interests

The authors declare that they have no competing interests. 

## Figures and Tables

**Table 1:**
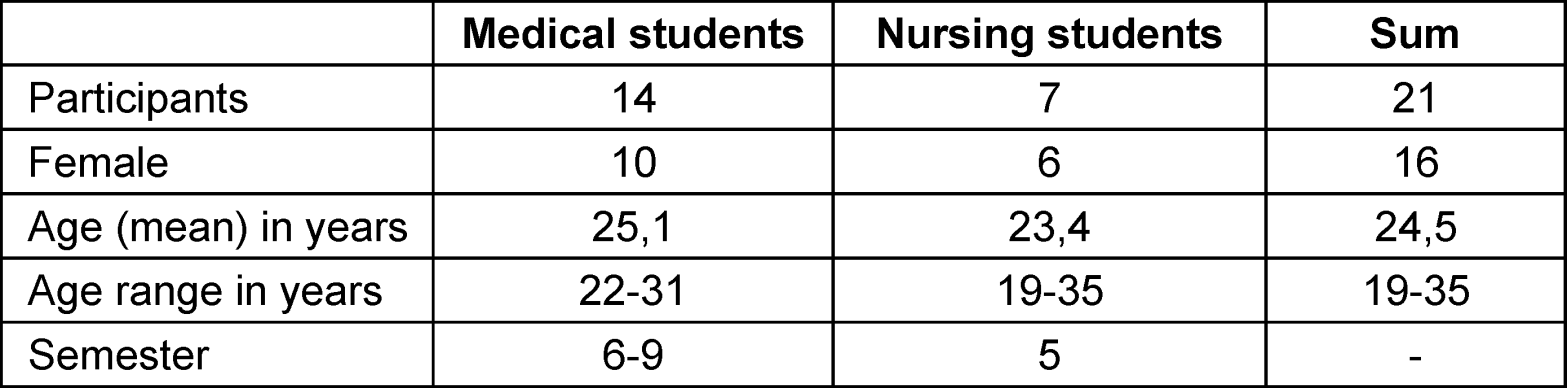
Characteristics participants

**Table 2:**
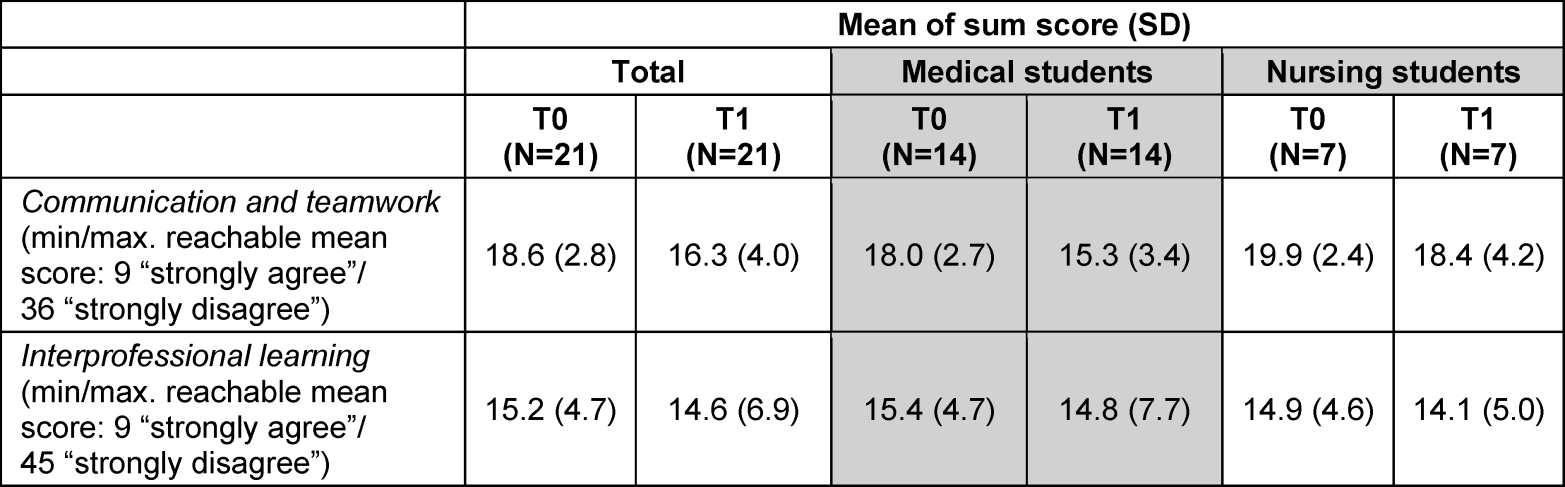
Change in UWE-IP-D

**Table 3:**
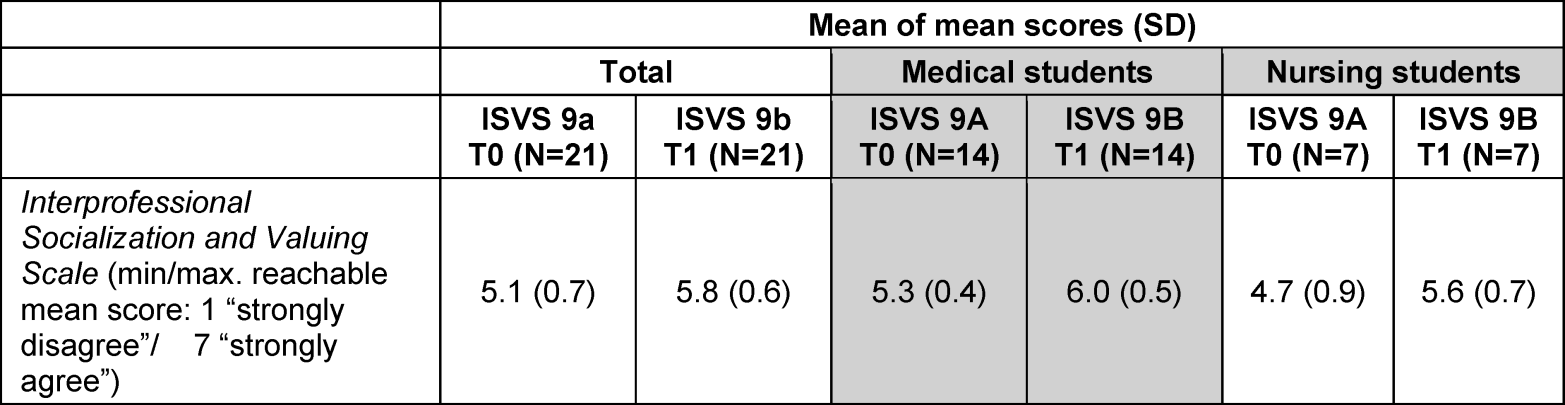
Mean of mean scores of ISVS 9A/9B at T0 and T1
